# Harnessing the promises of cell therapy, gene therapy, and regenerative medicine in Québec, Canada

**DOI:** 10.3389/fmed.2025.1581058

**Published:** 2025-06-03

**Authors:** Jean-Sébastien Delisle, Diane Fournier, Kim Santerre, Samuel Rochette, Ma’n H. Zawati, Maude Dumont-Lagacé, Simon Turcotte, Jason Robert Guertin, Patrick Vermette, Jacques P. Tremblay, Maxime Parisotto, Christian Beauséjour, Jean-François Gélinas, Louisa Petropoulos, Natasha Kekre, Stéphanie Michaud, Mélanie Dieudé

**Affiliations:** ^1^Centre de recherche de l’Hôpital Maisonneuve-Rosemont, Montreal, QC, Canada; ^2^Institut d’hématologie, oncologie et thérapie cellulaire (iHOT), Hôpital Maisonneuve-Rosemont, Montreal, QC, Canada; ^3^Département de médicine, Faculté de médecine, Université de Montréal, Montreal, QC, Canada; ^4^Québec Cell, Tissue and Gene Therapy Network, Montreal, QC, Canada; ^5^Transfusion Medicine, Héma-Québec, Montreal, QC, Canada; ^6^Centre of Genomics and Policy, Department of Human Genetics, Faculty of Medicine and Health Sciences, McGill University, Montreal, QC, Canada; ^7^Borealis Medical Writing Inc., Montreal, QC, Canada; ^8^Cancer Axis, Centre de recherche du Centre hospitalier de l’Université de Montréal, Montreal, QC, Canada; ^9^Department of Hepatopancreatobiliary Surgery and Liver Transplantation Service, Centre hospitalier de l’Université de Montréal, Montreal, QC, Canada; ^10^Axe santé des populations et pratiques optimales en santé, Centre de recherche du Centre hospitalier universitaire de Québec, Université Laval, Québec City, QC, Canada; ^11^Département de médecine sociale et préventive, Faculté de médecine, Université Laval, Québec City, QC, Canada; ^12^Centre de recherche en organogénèse expérimentale de l’Université Laval/LOEX, Québec City, QC, Canada; ^13^Laboratoire de bio-ingénierie et de biophysique de l’Université de Sherbrooke, Department of Chemical and Biotechnological Engineering, Université de Sherbrooke, Sherbrooke, QC, Canada; ^14^Faculté de Médecine et des Sciences de la santé, Centre de Recherche du Centre Hospitalier Universitaire de Sherbrooke, Sherbrooke, QC, Canada; ^15^Centre de Recherche du CHU de Québec-Université Laval, Québec City, QC, Canada; ^16^Département de Médecine Moléculaire, Université Laval, Québec City, QC, Canada; ^17^adMare BioInnovations, Montreal, QC, Canada; ^18^Centre de Recherche du Centre hospitalier Universitaire Sainte-Justine, Montreal, QC, Canada; ^19^Département de Pharmacologie et Physiologie, Faculté de Médecine, Université de Montréal, Montreal, QC, Canada; ^20^Centre C3i Inc., Montreal, QC, Canada; ^21^Division of Hematology, Department of Medicine, The Ottawa Hospital, Ottawa, ON, Canada; ^22^BioCanRx, Ottawa, ON, Canada; ^23^Axe immunopathologie, Centre de recherche du Centre hospitalier de l’Université de Montréal, Montreal, QC, Canada; ^24^Département de microbiologie, Infectiologie et immunologie, Faculté de médecine, Université de Montréal, Montreal, QC, Canada; ^25^Héma-Québec, Medical Affairs and Innovation, Montreal, QC, Canada

**Keywords:** immune effector cell immunotherapy, research funding, economic evaluation, ethics, biological drug approval, cell and tissue manufacturing

## Abstract

The last decade has witnessed tremendous progress in the fields of cell and gene therapy and regenerative medicine (CGT/RM). However, these advances came with their own challenges and opportunities, which may vary among jurisdictions depending on infrastructures, laws, regulations, access to funding, and socioeconomic, political and cultural aspects. In September 2023, the Québec cell, tissue and gene therapy network (ThéCell) and Héma-Québec held a symposium on the opportunities and challenges of CGT/RM in Québec, Canada. We collected and synthetized the views of scientists, CGT/RM manufacturers, business representatives, and experts in ethics and law, pharmacoeconomics, and regulatory affairs expressed during and after this symposium. Comments were grouped into seven strategic orientations: (1) a framework should be built for the development of CGT/RM products based on principles that will enable fair and timely access for all; (2) governments should spur private and public research investments in CGT/RM; (3) the skillsets of developers should be mobilized to foster the development and production of CGT/RM products in the academic and industrial settings, and the training of Québec’s workforce could be better aligned with industry and population needs to facilitate the industrialization of the sector, with the aim of reducing production costs and improving accessibility to patients; (4) Québec should ensure that the province’s production and healthcare capacity is aligned with current and future needs in CGT/RM products, considering the rapidly evolving landscape of CGT/RM; (5) regulatory awareness may be improved among developers through outreach approaches and early consultations; (6) the regulations governing the development of CGT/RM could be streamlined and adapted to the needs of these emerging products; (7) ongoing efforts to reform the clinical reimbursement framework could be continued in Québec’s public, single-payer healthcare system. This symposium provided guidance addressing current limitations and taking advantage of opportunities in CGT/RM in Québec. These considerations should help guide the development of new policies for CGT/RM products in Québec, keeping with principles of universal healthcare in Canada. We surmise that other jurisdictions face similar challenges, and the global CGT/RM community could benefit from these shared experiences to promote the optimal development and access to these promising therapies.

## 1 Introduction

The last decade has witnessed tremendous progress in the fields of cell and gene therapy (CGT) and regenerative medicine (RM), with several therapeutics entering routine clinical care. For example, chimeric antigen receptor T cell (CAR-T) therapies have shown unprecedented efficacy for several blood malignancies (1) and are now widely approved treatments in high-income countries (2, 3). Likewise, in 2023, the United States Food and Drug Administration (FDA) approved the first tumor-infiltrating lymphocyte (TIL) therapy for unresectable or metastatic melanoma and the first gene therapies for sickle cell disease (4–6). Other emerging CGTs will likely be approved in the coming years, including gene therapies for rare cancers and several monogenic conditions (7, 8). Such progress nurtures hope that many serious illnesses may soon be treatable, if not curable.

These seminal advances come with their own set of challenges (i.e., scientific, technical, economic, and regulatory) and opportunities. There are also wide differences between manufacturing processes and treatment modalities in what is considered CGT. For off-the-shelf allogeneic cell therapies (i.e., products that are not fully HLA matched to the recipient), it may be possible to standardize the source of starting cell material, and to manufacture products in larger batches. In contrast, allogeneic cell products that require a high degree of HLA-matching need to be manufactured specifically for the patient, thus incurring high front-loaded per-patient costs and longer lead-time before patients can be treated. This is especially problematic for cell therapies for rare diseases, as they target a smaller market that is less appealing to the private sector.

Similarly, while *in vivo* gene therapies may be manufactured in larger batches and avoid *ex vivo* cellular or tissue processing altogether, one must always consider the intrinsic quality of the targeted cell that is altered for therapeutic purposes. Autologous CGT products must often address important challenges due to the heterogeneity of the starting material. Indeed, depending on the medical history of a patient (e.g., prior rounds of chemotherapies or radiation), the quality and/or quantity of starting material can vary significantly from patient to patient, leading to variable product quality.

Traditional pharmacoeconomic approaches may not adequately assess the true value of these treatments. Contrary to traditional drugs, such therapies may have the potential to offer substantial benefits that could last years, if not decades. However, we still do not fully know the long-term efficacy of CGT, especially given that each therapy is different.

Although these challenges are universal, their impacts and proposed solutions vary across jurisdictions. In Canada, health care is provided through a single-payer, universal system whose funding, regulation, and management involves two levels of government: the federal government regulates drug approvals and reviews cost-effectiveness (outside the province of Québec); and the provinces provide the majority of funding, determine which drugs are reimbursed, and manage day-to-day operations (in Québec, a provincial agency [i.e., the *Institut national d’excellence en soins et en services sociaux*] reviews cost-effectiveness and informs drug reimbursement policies). Both the federal and provincial governments provide research support. Because the provinces are responsible for delivering health care, they also have a prominent role in shaping the CGT and RM ecosystems. Of note, public provincial health plans cover all drugs administered in hospitals, but not other prescription drugs administered outside hospitals. In Québec, the coverage of out-of-hospital expenses is diverse, ranging from public pharmacare to private, employer-sponsored drug plans. While public research funding comes largely from the federal government, the provinces have a prominent role in scientific and economic development (which they share with the federal government), and in shaping the CGT and RM ecosystems.

Québec boasts a number of assets to spur the development of a thriving CGT and RM sector but also faces a number of challenges. The province hosts a vibrant biotechnology sector and top-tier universities, as well as larger biopharmaceutical companies (e.g., Moderna, Bausch Health). However, as is the case in many other high-income countries, the province faces a severe shortage of highly qualified personnel (HQP), including workers specializing in CGT and RM. Québec’s health care system is also in a particularly dire state due to operational challenges, an aging population, and the aftermath of the COVID-19 pandemic.

In September 2023, a virtual symposium was held on the opportunities and challenges of CGT and RM in Québec. The symposium was organized by Québec’s cell, tissue and gene therapy network (i.e., ThéCell; a provincial network of >150 researchers in CGT and RM) and Héma-Québec (i.e., Québec’s blood, cell, and tissue [BCT] provider). The event brought together scientists, physicians, and experts in law and ethics, pharmacoeconomics, regulations, and drug reimbursement and business development, who shared their views of Québec’s ecosystem in CGT and RM. This article synthesizes and expands on the discussions held during and after this symposium, with the aim of guiding the development of policies that will help bring new CGT and RM products to patients.

## 2 An ethical framework for cell and gene therapy and regenerative medicine

In collaboration with Canadian and international experts, McGill University’s Center of Genomics and Policy, under the leadership of Prof. Bartha Maria Knoppers, developed a Charter for RM (9). The Charter offers a high-level vision for the future of RM that recognizes its advantages. It sets overarching principles to guide the development of an ethical framework for this emerging discipline, which ultimately aims at facilitating the conduct of research. Notably, the Charter is based on shared ethical norms and is therefore an opportunity to establish an international consensus on human rights in RM research.

Three rights underlie the Charter: the right to science, the right to health, and the right to non-discrimination (9). The right to science “includes freedom and rigor of scientific inquiry, the duty to provide an enabling environment for responsible science, and the right of everyone to benefit from scientific advancement” (9). The right to health “mandates that healthcare services, goods, and facilities be available, accessible, and of good quality” (9). Lastly, the right to non-discrimination “entitles all humans to equitable access to preventative and therapeutic health services” (9).

Balancing these rights with established principles (e.g., safety, transparency, integrity) and existing constraints (e.g., financial) will be challenging. Historically, for example, ethics review boards have prioritized rights other than the right to science, even though this right is widely recognized among ethical experts. Indeed, article 27 of the 1948 Universal Declaration of Human Rights enshrines the right of everyone to “[…] share in scientific advancement and its benefits” (10). Furthermore, article 15 of the 1966 International Covenant on Economic, Social and Cultural Rights identifies the right to “enjoy the benefits of scientific progress and its applications” (11). Ethics review board members may benefit from training sessions to increase their awareness of research limitations and patient risks in the emerging fields of CGT and RM.

The sections below discuss how these rights provide guidance for addressing current challenges and taking advantage of opportunities in CGT and RM, while considering regulatory, logistical and commercial imperatives.

## 3 Promoting the right to science

Several issues require attention from all stakeholders and policy makers to uphold the right to science in the CGT and RM space. The panelists discussed many considerations that pertain to this right, including funding, intellectual property, scientific expertise, Québec’s production capacity, ethical considerations, regulatory issues, and the burden of early-phase trials.

### 3.1 Public funding

In Canada, public research funding mostly hinges on federal agencies and programs, although some provincial agencies also offer strategic and complementary financial support. However, according to the participants, the traditional funding agencies (e.g., Canadian Institutes of Health Research [CIHR], Natural Sciences and Engineering Research Council of Canada) mostly support fundamental science and reward researchers based on academic performance metrics, such as number and/or high-profile publications. As a result, funding for process developments, such as scale up or automation, is difficult to obtain through traditional agencies. Indeed, this type of research is more difficult to publish (preservation of trade secrets, misperception as less innovative, etc.), provides little training opportunities, requires considerable resources, and is not considered novel science by most grant panels, even though feasibility science can help acquire knowledge to bring CGTs to the clinical space. Therefore, the current public funding system does not reward the development of a product toward an end use. This partly explains why many products do not reach patients in a timely fashion or, worse, are never even tested in patients. Too often, academic researchers are evaluated based on their publication record and the funding they receive rather than their projects’ milestones and deliverables.

This being said, several early-phase clinical trials in CGT and RM were recently funded — both through regular competitions and special initiatives. For example, in Spring 2022, the CIHR announced $250M in new trial funding through the Clinical Trials Fund. A key aim of this measure is to support high-priority research areas that align with Canada’s Biomanufacturing and Life Sciences Strategy, including “Clinical trials in emerging technology areas (e.g., novel vaccine platforms, cell and gene therapies) with high potential to solve current and future health challenges” (12).

Furthermore, the importance of translational (“bench-to-bedside”) research is recognized by federally funded networks, such as the Stem Cell Network, which invest in translational and early-phase clinical research. Another noteworthy example of a funding body focused on translational research is BioCanRx, which invests in proof-of-concept clinical studies, pre-clinical development, process development, and clinical trials. Finally, the Canadian Foundation for Innovation (CFI) and the provincial government contribute to increasing Québec’s biomanufacturing capacity through grants for equipment and infrastructure, which is also in keeping with Canada’s Biomanufacturing and Life Sciences Strategy. Along with direct support to research centers, scholarships, career awards and targeted grant funding, the Fonds de recherche du Québec (FRQ) is a vital actor that promotes networking in CGT and RM. However, the two levels of government offer limited perennial support to maintain and modernize the manufacturing facilities that are required to sustain the growth of the CGT and RM sectors.

### 3.2 Private funding

Many panelists pointed to the lack of adequate funding for large — and costly — trials as a key barrier to bringing new CGT and RM products to the domestic market. Indeed, many panelists indicated that Québec lacks sufficient sophisticated venture capital and private investments to launch such trials or support adequately its biotechnology sector. Many (although not all) local institutional investors are guided by an “exit strategy” that does not prioritize the local development of discoveries up to the clinical stage and the establishment of perennial businesses that will serve the province’s economic interests. Further, some invest in companies headquartered outside Canada, although many of those that receive public funding must comply with state-mandated quotas of investments in local businesses. This challenge, however, is not unique to Québec: Canada as a whole has been grappling with anemic R&D spending in the private sector for years, with no signs of progress (13).

This limitation may be alleviated by several means. As argued in a recent review, public-private partnerships (PPP) may help spur the development of new products by leveraging the strengths of academia and biopharmaceutical companies (14). This review also highlights the fact that these issues are not unique to Quebec or Canada, but are challenges faced worldwide in the CGT and RM field. The private sector partner can help scale-up the production of a CGT or RM product and acquire market authorization in multiple jurisdictions, whereas the academic partner may be better positioned to develop products viewed as too risky by the private sector, for example because they target a relatively rare disease entity (14). An example of a Canadian PPP is the federally funded “Business-Led” Networks of Centres of Excellence (NCE) and the Centre of Excellence in the Commercialization of Research programs — both sunsetted NCE programs that nonetheless established strong organizations, including some supported by the Strategic Science Fund until 2032.

The NCE model may be expanded to provide CGT and RM developers with readily accessible tools to support innovation. These may include support for the development of manufacturing tools and facilities for the production of CGT and RM products, which are supported by the provincial and federal governments. Such innovations could provide key reagents at an affordable cost for academic projects and emerging biotechnology companies. One example of such a tool would be a Canadian platform able to handle induced pluripotent stem cells and other engineered cell types, an initiative that is being considered by the National Research Council Canada. Automation may be another mean of reducing costs and may significantly increase production given the labor shortage in Québec. Moreover, the lack of venture capital may be addressed (at least on the short term) by doubling down on existing organizations that assist and/or invest in local biotechnology companies. These include government-funded venture capital funds, such as adMare BioInnovations, the healthcare venture fund of the Business Development Bank of Canada, the Fonds de solidarité FTQ, and the Caisse de dépôt et de placement du Québec, as well as private funds. In addition, cell production facilities may partner with and invest in local companies, as done by C3i (the largest good-manufacturing-practices [GMP]-compliant cell production facility in Canada) in three Canadian-based companies.

### 3.3 Intellectual property

Another challenge is the acquisition of patents. The symposium panelists agreed that Québec universities lack the financial resources to patent new discoveries. Specifically, although most universities are willing to acquire patents, many lack funding to preserve them beyond 2–3 years, which is often insufficient to find a suitable business partner. In particular, many smaller universities also lack the expertise and business intelligence capabilities to build a strong intellectual property strategy.

### 3.4 Expertise and training

**FIGURE 1:**
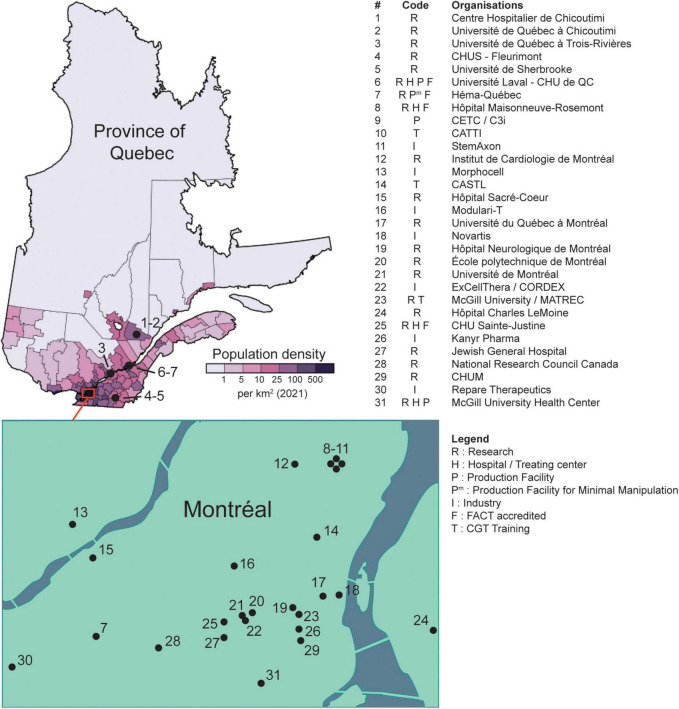
Locations of public and private organizations involved in CGT and RM (non-exhaustive list). Universities and hospitals where researchers affiliated to the ThéCell network are located are labeled with “R”; clinical centers performing CGT (e.g., stem cell transplantation, or CAR-T) are labeled with “H”; privately held companies developing CGT are labeled with “I”; production facilities refer to biologics manufacturing centers compliant to GMP standards and are labeled with “P” or “P^m^” for production facility for minimal manipulation. CHUS, Centre Hospitalier de l’Université de Sherbrooke; CHU de QC, Centre Hospitalier Universitaire de Québec; CETC, Centre d’Excellence en Thérapie Cellulaire; C3i, Centre for Commercialization of Cancer Immunotherapy; CATTI, Canadian Advanced Therapies Training Institute; CASTL, Canadian Alliance for Skills & Training in Life Sciences; MATREC, McGill Advanced Therapies Research and Education Center; CHUM, Centre Hospitalier de l’Université de Montréal; FACT, Foundation for the Accreditation of Cellular Therapy; CGT, Cell and Gene Therapies. The population density map taken from https://en.wikipedia.org/wiki/Quebec#/media/File:Quebec_Population_Density_2021.svg (distributed under a CC BY-SA 4.0 license, see https://creativecommons.org/licenses/by-sa/4.0/).

According to the panelists and other invited experts, Québec stands out for its capacity to innovate in the field of cell and gene therapy, with several university and hospital centers developing CGT technologies at the R&D stage. A non-exhaustive list of public and private institutions involved in CGT and RM and their geographical location are provided in Figure 1. The expertise needed for the development of CGT and RM products is abundant in Québec, although the sector faces the same labor shortage as many other industries throughout the province. However, one of the key conclusions arising from the discussions at the Symposium was the clear need for a harmonized development strategy for CGT and RM in Quebec that would allow identification of priorities for an efficient management of resources to close the existing gap between the R&D stage toward commercialization.

Indeed, the province’s expertise is not properly mobilized, with many experts working in isolation. Therefore, new initiatives are necessary to integrate those skill sets, both in the academic and industrial sectors. This integration may be enabled by existing organizations, such as academic networks (e.g., ThéCell research network), non-profit research organizations (e.g., Mitacs), and non-profit research consortiums, which could liaise with industry partners and academic investigators to share complementary expertise. Moreover, several provincial and federal funding programs have encouraged academic-industry partnerships, monitoring project advancement to ensure the timely achievement of milestones and production of tangible deliverables as seen in industry. Such projects offer opportunities for trainees and research personnel to integrate the skills required to operate in applied research and in the private sector. However, in Québec, there is a wide gap between the needs of traditional discovery research in an academic, single-investigator-led setting and product-oriented research involving multidisciplinary teams. Several argued that a significant overhaul is required for the academic sector to adapt and meaningfully contribute to the delivery of CGT and RM products.

Another issue raised during the symposium is that many college and university students are ill-prepared to join the workforce upon graduating, hence the need to reform their training. For graduate university students, the scope of their training could be expanded to better integrate them into the workforce. For example, some panelists raised the possibility of including finance and leadership training in the curriculum of graduate students, which would be valuable to academic as well as industrial researchers. One panelist suggested that the COOP program offered by the Université de Sherbrooke, which provides students with three compensated four-month internships, serve as an inspiration to other universities for providing hands-on training to university graduates. As for vocational school students (i.e., collège d’enseignement général et professionel [CEGEP]), they may benefit from more opportunities to acquire hands-on training with good laboratory/manufacturing practices (GLP/GMP). Some panelists also brought up the training sessions offered by non-profit organizations, which would be of interest to many students and researchers. For example, the International Society for Cell and Gene Therapy offers accredited training programs that cover regulations, leadership, and hands-on laboratory skills (among other things). Some organizations (e.g., the Canadian Advanced Therapies Training Institute and the Canadian Alliance for Skills and Training in Life Sciences) also offer online and onsite training sessions on a wide range of topics, including regulations, and GMPs and their underlying principles. Another noteworthy example is CanPRIME 2.0, a program cofunded by BioCanRx and Mitacs that offers college and university students a 9-month, paid internship in a GMP facility.

Besides universities, the role of CEGEPs was also discussed. These vocational schools are increasingly integrated in Québec’s industrial sector and play a vital role in the training of HQP. From the perspective of some panelists, CEGEPs excel at simulating the work environments that their graduates are poised to encounter on the job market. Universities may view at least some aspects of CEGEP training as an inspiration to better integrating their graduates in the work force. Of note, there are three distinct organizations in Montreal that provides industry-informed technical training specializing in biopharmaceutical manufacturing (see Figure 1).

Although automation holds promise to alleviate the shortage of HQP, it also entails its own set of challenges. The personnel will require training to handle these new automated tools. Additionally, at least some workers will be needed to carry out routine maintenance tasks and repairs. These considerations should be factored in before implementing automated tools.

Another issue is that not all developers — especially those in academia — have prior experience with the operational challenges and regulations that govern the production of CGT and RM products for early-to-late-stage clinical trials, or even commercialization. Canadian cell production facilities may be key to acquiring this experience. Recently, Health Canada granted C3I a drug establishment license for the commercial production of cell therapies — a first for a Canadian contract development and manufacturing organization (15). With the achievement of this milestone, the organization is well positioned to significantly expand its production in the coming years.

At least part of this experience may also be offered by BCT providers, such as Héma-Québec. Indeed, BCT providers have unique, long-lasting connections with their national regulatory bodies and often adhere to numerous voluntary accreditations that enable a sustainable distribution of substances of human origin (SOHO), locally and internationally. They also have important assets to act as regional competence centers in the development and manufacturing of CGT and RM products (16, 17). Their existing infrastructures (e.g., collection centers, including apheresis, processing facilities for peripheral stem cells, and cord blood cryopreservation and storage), services (e.g., donor recruitment, stem cell donor registry, research and development capabilities, and specialized laboratories), and medical expertise (e.g., donor eligibility) could also be leveraged for the production of new products, including CGTs. Héma-Québec also distributes tissues (but not solid organs) throughout Québec — a SOHO that may also be leveraged to develop RM products.

In other countries, the expertise of BCT providers has in fact proved critical in developing many CGTs. For example, a recent review article described the key role of Sanquin (i.e., the Dutch blood service) in the production of TILs in the Netherlands and Denmark (see section “Promoting the right to health” for more details) (16). Similarly, the same review described the roles of Banc de Sang I (Teixits, Spain), Centre Hospitalier Universitaire Liege (Liege, Belgium), and University Hospital of Montpellier (Montpellier, France) in the development of commercial CAR-Ts (16). As of this writing, Banc de Sang I is involved in four ATMPs being investigated in academic research and nearly 20 ATMPs being investigated in clinical research (18). Lastly, NMDP (formerly known as the National Marrow Donor Program and Be the Match) leveraged its infrastructure and expertise to deliver safe hematopoietic stem cell (HSC) products, thereby expanding its role in facilitating the supply and distribution of CGTs to patients.

### 3.5 Production capacity

Québec currently has a suboptimal capacity to produce GMP-compliant drugs for clinical trials (even for smaller pre-clinical studies), which hampers the right to science. This also limits the ability of contract research organizations to conduct preclinical studies in the province.

To address this limitation, the province may consider doubling down on existing (publicly funded) production facilities to foster the burgeoning of its CGT and RM ecosystem. Indeed, Québec is home to C3i — the largest GMP-compliant cell manufacturing facility in Canada (see Figure 1). Such a centralization would allow for economies of scale, thereby reducing manufacturing costs. Besides C3i, some production facilities have been funded by the federal government and may also contribute (see Figure 1).

However, given Québec’s vast territory, dispatching fresh products from a single production facility throughout the province might prove challenging. Distances bring logistical challenges especially in a territory that is as vast as Quebec with a very thinly spread population (see Figure 1, also applies for other Canadian provinces). Indeed, bringing donors or patients to clinical centers and housing them for the prolonged periods needed for many CGT also implies a high social cost. Although inevitable when specialized care is required, one may predict that increasingly transportation or cryopreservation of starting materials (i.e., cells from donors) and/or final products and decentralized treatment of patients will reduce the cost of CGT and provide innovative therapeutics to underserved and remote populations. This issue might be addressed through a partnership with Héma-Québec (the provincial BCT provider), which routinely transports many types of products throughout the province and internationally from two processing facilities located in Montreal and Québec city. Of note, other centers in Québec have experience in shipping CGT/RM products throughout Canada (e.g., LOEX/CHU de Québec-Université Laval, Figure 1) and abroad (e.g., C3i).

The relatively small number of CGT and RM trials conducted in Québec may be because the province has yet to demonstrate its ability to commercialize (locally and abroad) CGT and RM products. One panelist suggested that, once the patents of current CGT and RM products expire, Québec may consider developing a biosimilar to showcase the province’s know-how in the production of CGT and RM products. Although such a project would also require the conduct of a demonstration study, it may alleviate some concerns among industry stakeholders and help spur additional investments in more risky endeavors.

### 3.6 Ethical considerations

It was also signaled that ethical aspects are often considered too late in the planning of clinical trials. In fact, being able to think about the possible challenges or issues that would be identified by bodies overseeing the review of an application (i.e., Research Ethics Boards) and addressing them early on will ultimately save time by reducing the back and forth in communications and review. Of note, policy makers could simplify the conduct of trials across many provinces: currently, developers must obtain multiple ethics approval (one per institution) — a time-consuming process that could be avoided if some institutions allowed a multisite, coordinated ethical review. Currently, such multisite reviews are allowed among institutions located within Québec, but not for projects involving Québec and out-of-province sites.

### 3.7 Regulatory issues

The current regulatory landscape for CGTs is highly complex and offers no one-size-fits-all approach. Developers must, therefore, leverage their product knowledge (and, possibly, the assistance of regulatory intelligence professionals) to develop a regulatory strategy that is adapted to their product and its mechanism of action. For example, a strategy might include the development of *in vitro* assays for the quality control and potency assessment of CAR-T cell products, with a view to assessing cell proliferation and determine whether the product’s anti-tumor efficacy correlates with post-manufacturing proliferative capacity (19). Of note, a key consideration that is unique to CGTs is the potential carcinogenicity associated with *ex vivo* cell expansion, especially with genetically modified cells.

Developers and regulators may tackle regulatory challenges by various means. Developers should seek the input of regulators early on to quickly grasp the extent of testing that their product must undergo, which will help prevent unforeseen delays and reduce costs during the pre-clinical and clinical development phases. These early consultations may even help secure funding as regulatory compliance is a top priority for investors and public funders. They should also align their regulatory strategy with their business strategy, for instance, by assessing the regulatory frameworks in jurisdictions of interest and laying out a strategy to secure approvals in multiple jurisdictions. Regulators, on their end, may strengthen their engagement with the scientific community by launching initiatives to ensure their processes are correctly understood by everyone, especially first users. For example, the FDA reaches out to the scientific community by sending representatives to attend and present at scientific meetings.

While most national bodies follow ICH guidelines which provide a foundational framework for drug development requirements, significant differences in each country’s legislation can complicate the streamlining of approvals across jurisdictions. This is especially true for rare diseases where the targeted patient population is small but has substantial medical needs. Regulatory agencies from different countries may have varying concerns and interests regarding specific CGT products influencing what is considered sufficient evidence to support drug product claims. In addition, as clinical practices can differ between countries, differing definitions of what constitute the standard-of-care can complexify obtaining protocol approval for one specific confirmatory (phase 3) clinical trial across multiple jurisdictions. Given the high costs of drug development, it is particularly challenging to develop a strategy that ensures streamlined approvals across multiple jurisdictions at the lowest possible cost.

Recognizing these challenges, some regulators offer programs and regulatory pathways that streamline and support the approval of promising medicines (e.g., the PRIME scheme of the European Medicines Agency; the fast track, breakthrough, and RM advanced therapy designations of the FDA). Currently, such programs are absent in Canada, but the Food and Drugs Act was amended in 2019 to provide the regulator with more flexibility to evaluate advanced therapeutic products. Moreover, in 2022, Health Canada initiated consultations regarding its regulatory framework for advanced therapeutic products and has recently started a formal consultation on point-of-care manufactured CAR-T products. The agency also offers consultation meetings before a formal clinical trial application, new drug submission, or priority review submission, during which developers can present data and discuss drug development concerns.

The panelists also brought up the low awareness of GMPs of many developers as a recurrent problem hampering clinical development. Indeed, early adherence to GMPs ensures protocol robustness, expedites clinical development, and reduces costs. Developers with less experience may benefit from training sessions, mentoring, and even financial support from organizations that partner with local pharmaceutical companies (e.g., Startup studio model).

Non-trial access programs also deserve consideration given patients’ growing interest and their potential impact on research. Health Canada’s “special access program” is the Canadian counterpart of the FDA’s “expanded access” program: both are regulated, non-trial access programs that offer seriously ill patients the possibility of receiving investigational drugs rapidly while retaining some protections (20, 21). Of note, however, there is no Canadian counterpart to the United States “right to try” legislation, through which patients may access investigational drugs without FDA authorization. It would be surprising to see such a legislation enacted in Canada at this time given that the benefits of the “right to try” legislation remain controversial (21). For products at an early stage of development, an alternative to non-trial access programs that is often favored by regulators is the so-called “single-patient trial” (a.k.a. open-label individual patient study in Canada), which offers the rigor of clinical trials with everything it entails in terms of time and resource investments by sponsors (22).

Despite their appeal, a chief concern of non-trial access programs is that they may divert resources — including patients — away from trials (21). This limitation is further compounded for CGT and RM products, which often target small patient populations. On the other hand, such programs could allow for the early collection of real-world evidence and clinical data that may not otherwise be collected in a trial (21). Furthermore, some patients may receive and benefit from a drug later proven safe and efficacious (21). Readers interested in knowing more about the practical and ethical aspects of non-trial access programs (outside Canada) are referred to other excellent review articles (21, 23).

### 3.8 Regulatory, financial, and logistical burden of early-phase trials

Realistically, overcoming the above barriers may take years. In the meantime, many patients have pressing medical needs that may remain unaddressed, but the regulatory, financial, and logistical burden of early-phase trials hinder research and, by extension, patients’ access to potentially life-saving drugs.

To expedite the conduct of early-phase trials, some panelists proposed that regulators show increased flexibility, adapting their regulations to the needs of early-phase trials. Although this proposition raises ethical questions and was controversial among the panelists, the putative benefits would likely be significant, as the successful conduct (or initiation) of early-phase trials may facilitate the acquisition of funds for more costly advanced-phase trials, especially if the preliminary results are promising.

A factor that contributes to the financial burden of early-phase trials is that the current reimbursement policies in Québec and other provinces sharply distinguish routine clinical care from clinical research. As a result, all research-related costs — which sometimes include standard-of-care drugs used in the control arms of trials — are borne by the study sponsor, which can be detrimental to the conduct of smaller trials supported by institutions or academic grants. To favor innovation and patient access to novel therapies, provincial governments should consider reimbursing the clinical costs of patients who enroll in trials. In the United States, these costs are covered by insurance companies, thereby alleviating the financial burden of clinical trials.

The logistical burden of early-phase trials in CGT and RM is another significant barrier. Trials often have heterogeneous protocol requirements regarding the shipping conditions, thawing instructions, and testing of the cellular starting material (24–26). This makes it difficult for health care centers to comply with them, especially when a given product is infrequently handled by center personnel. The health care centers participating in early-phase trials may consider discussing with the sponsor to streamline and standardize the protocol prior to trial launch (24, 27). This logistical burden is further compounded by differing contracts between clinical sites and sponsors, between manufacturing sites and sponsors, and quality agreements. Harmonizing these administrative considerations would greatly alleviate the burden of conducting trials in Canada. Of note, in Québec, the current labor shortage and the limited resources of many health care centers may add to these logistical challenges.

Clearly, the current situation severely hinders patients access to early-phase trials in CGT and RM in Québec. As such, financial, legal, regulatory, and logistical burdens limit the conduct of early-phase trials in Canada and were identified as a key barrier not only to the right to science, but also to the right to health.

## 4 Promoting the right to health

Notwithstanding the aforementioned considerations pertaining to the right to science, patient care could be improved by enhancing access to approved therapies. In this regard, Canada’s performance has been lackluster. For example, in some provinces, 7 years have elapsed between the approval of the first CAR-T and the first patients receiving it.

Therefore, patients’ right to health may be promoted by ensuring CGT and RM products are accessible faster after their approval, while promoting a cost containment philosophy. The best approach to achieving this goal may be decided upon after seeking the input of patient partners and the broader community, while considering the following factors: production and distribution, the costs to the public system, and the parameters that ought to be included in economic evaluations.

### 4.1 Production and distribution

Québec hosts top-tier universities and the largest GMP-compliant cell production facility in Canada (Figure 1), but more investments will be needed as new therapies are approved. As mentioned above, Québec’s production capacity may hamper the right to health if it cannot meet the future demand for approved CGT and RM products.

To better balance the supply of and demand for CGT and RM products, Québec may therefore consider exploring production models implemented elsewhere, such as the Catapult Network for CGT in the United Kingdom (UK). Specifically, the CGT Catapult supports UK developers of advanced medicinal therapy products (ATMPs) through the entire drug commercialization process, from research and development to patient access through the public National Health Service (NHS) (28, 29). Should such models improve Québec’s production and delivery capacity, at least some of it may be allocated for research needs. This way, patients who are ineligible to receive commercially available (and reimbursed) products may still receive potentially life-saving medications or CGT/RM products.

The production and distribution of CGT and RM products is complex and challenging relative to traditional drugs, hence the variable access to these therapies in Québec (and Canada). To address this issue, Québec and Canada might consider conducting clinical trials with a view toward increasing the capacity and efficiency of biomanufacturing and product distribution. One such trial is the Canadian CLIC-01 trial, which is part of national and provincial efforts to provide access to CGT and RM via clinical trials. CLIC-01 is a single-arm, open-label phase I/II trial that aimed to assess the feasibility of providing a CD19 CAR-T cell therapy produced at a local manufacturing facility (30). The final product (CT-1901) was highly efficacious and was infused within ∼2 weeks after apheresis, a notable improvement compared with the 4–6 weeks typically required for the infusion of commercial CAR-Ts (30). This achievement is especially remarkable given the distance between the manufacturing facility and the CAR-T cell administration sites (i.e., > 4300 km apart). Secondary benefits that stem from this production model include (1) the training of a workforce that is competent in CGT and the administration of these products, (2) the local biomanufacturing of these products, and (3) savings to the health care systems due to the lower costs associated with CT-1901.

Other countries have explored similar modes of production. For example, Denmark and the Netherlands publicly funded a randomized controlled trial for patients with metastatic melanoma. The trial compared an immune checkpoint inhibitor with a TIL immunotherapy manufactured at Sanquin (the Dutch blood service) and other on-site, in-hospital cell laboratories (7). The manufacturing process was adapted from that initially developed by the National Cancer Institute (NCI) in the United States (31). The efficacy and safety of these cells were, as anticipated, in line with those reported by the NCI, and superior to those of the immune checkpoint inhibitor (7, 31). Based on these efficacy results and other cost-effectiveness analyses, Denmark and the Netherlands approved TIL for this indication, initially under a hospital exemption.

However, Québec has finite production resources, and so not all CGTs and RM products may be produced domestically. Thus, the province might need to strategically select the treatments that should be produced locally based on objective criteria, such as patient needs and costs. For example, gene therapies for diseases that are particularly prevalent in Québec (e.g., myotonic dystrophy type 1, oculopharyngeal muscular dystrophy, tyrosinemia type I, autosomal recessive spastic ataxia of Charlevoix-Saguenay) may be prioritized over other ones that are likely to be produced elsewhere. Similarly, treatments for diseases that progress rapidly (and thus require very rapid treatment initiation) could be prioritized for local production, as shown in other jurisdictions (e.g., Spain) where point-of-care CAR-T cell manufacturing facilities have been proposed to reduce delays between cell collection and infusion. Producing these strategic treatments domestically also reduces the risks associated with international supply chain disruptions, which have been exposed by the COVID-19 pandemic. The domestic production of new, investigational CGT and RM products may be enabled by existing cell processing facilities that have the necessary accreditations (e.g., FACT, see Figure 1) and infrastructures to take on this responsibility (32).

However, some countries may choose not to develop a cell manufacturing capacity, cover associated research and development costs, and provide a regulatory pathway for the approval and reimbursement of medical innovations developed along an open science model. These countries may instead consider commercial products like lifileucel, which has recently become the first FDA-approved TIL product (4). However, the prohibitive cost of lifileucel — which currently sits at > 500,000 USD per treatment (33) — may be a strong deterrent to a nationwide public coverage.

Québec may also need to plan for the eventual expiration of some patents in CGT and RM, notably those for CAR-Ts. This may be an opportunity for existing generic drug companies and publicly funded cell production facilities to expand their product offering. It may even spur the creation of new generic drug and biosimilar companies specializing in CGT and RM. Initiating consultations with government and industry stakeholders may prove instrumental to lay out a detailed strategy.

However, regardless of the chosen approach to boost Québec’s production capacity, manufacturing should be designed and implemented to improve productivity and reduce costs with sound engineering principles and a lean supply chain management.

### 4.2 Treatment costs

The current cost associated with CGT and RM products may not be sustainable if these therapies were to become the standard of care for common diseases, although costs may be reduced with economies of scale and a better optimization of infrastructure, equipment, and personnel. However, using such treatments for rare diseases may be affordable given the small size of these populations (when taken individually), although the indications of CGT and RM products target increasingly large populations (e.g., CD19 CAR-T in autoimmune diseases) (34). Additionally, philanthropy may help offset at least part of the costs of CGTs, not only for infrastructure development but also for clinical programs.

### 4.3 Pharmacoeconomic considerations

Although the demand for CGT and RM products is poised to grow, there is a risk of funding production technologies that may be outcompeted by others that are more effective or less costly (or both). Such a risk could be mitigated through projections and risk analyses, hence the importance of further developing a highly skilled economic expertise in Québec.

Another important consideration is that, despite progress, the current economic evaluation framework is largely based on small molecules and biologics (although other CGTs and RMs are increasingly used as comparators). However, unlike small molecules and biologics, CGT and RM products bear heavily front-loaded costs and may cure many conditions that currently remain chronic or fatal (35). Moreover, their long-term benefits are currently uncertain (35) and will likely remain so for a while, hence the need to continually evaluate their effectiveness and cost profiles.

These uncertainties may be addressed by more flexible reimbursement policies. For example, developers could be (partially) reimbursed early — before a formal demonstration of long-term effectiveness — possibly by making future reimbursements contingent on the treatment meeting its promised effectiveness (36). However, such an outcome-based reimbursement model may be ill-adapted to Québec’s ecosystem, which is dominated by relatively small biotechnology companies and startups that often rely on a single revenue stream. Indeed, these smaller companies may be unable to afford the risk of not being paid the full asking price for their products, especially given the small size of the potential recipient populations (e.g., rare monogenic diseases).

In addition to cost and effectiveness considerations, reimbursement decisions are increasingly contingent on the (limited) operational capacity of Québec’s health care system. CAR-Ts, for example, require that patients be hospitalized 7-10 days after receiving an infusion (37). This stay can last 17 days for TIL recipients (7) and up to 42 days for recipients of genetically modified HSCs (38). Clearly, these hospital stays will further pressure Québec’s overburdened health care system, which is why they are considered in current reimbursement decisions.

Another noteworthy aspect concerns the hybrid drug coverage in Canada, whereby drugs are covered by provincial health plans only insofar as they are administered in a hospital. Therefore, some drugs currently covered by public health insurance plans might no longer be covered if manufacturers begin offering more convenient, home-based treatments.

### 4.4 Accreditations

Health care organizations may also help uphold patients’ right to health by obtaining recognized accreditations that will help them keep abreast of developments in CGT and RM. Such accreditations may help select the organizations with the necessary infrastructure and quality management system to handle some or all of the steps in the manufacturing of new CGT and RM products (32). For example, the Foundation for the Accreditation of Cellular Therapy (FACT) awards a voluntary accreditation that testifies to compliance with comprehensive standards (39). FACT also recently launched a Consulting service for audits and assessments to assist CGT professionals. In Québec, many transplant programs, apheresis centers, and processing facilities are FACT-accredited and share best practices among colleagues and centers. Accredited organizations (Figure 1) may consider forming a reference network to further promote knowledge sharing, which would also benefit unaccredited centers.

## 5 Promoting the right to non-discrimination

The emergence of CGT and RM products might raise difficult (but unavoidable) ethical questions regarding the right to non-discrimination.

Given their prohibitive cost, governments may be tempted to prioritize certain patient populations. A donor’s age and overall health condition, for example, might be factored into the decision to reimburse these treatments. While unsettling, these considerations are routinely applied in solid organ transplantations — a field that may inspire the development of an ethical framework for the prioritization of CGT and RM products.

Access to CGT and RM products may also be unequitable among patients due to factors beyond the control of decision makers. As mentioned above, patients with rare diseases may reap little benefit from scientific advancements, as rare diseases remain less lucrative than common ones for developers. Similarly, among patients with rare hereditary diseases, those with uncommon mutations may not be prioritized by drug developers. Further, even when a drug becomes available for these underserved populations, a developer’s asking price may be too high for the fiscal capacity of Québec’s health care system.

Geographic considerations are also of prime importance, especially in a vast, sparsely populated country like Canada. For example, patients who live in remote areas face an additional barrier to accessing CGT and RM products, because these therapies must be administered in specialized centers. Moreover, very few centers in Canada conduct CGT and RM trials, and the limited number of vacancies is generally filled by urban residents. Future studies should explore how distance from a treatment center impacts access to CGT and RM products and long-term outcomes.

Furthermore, some investigational allogeneic medicinal products have more complex compatibility requirements (e.g., human leukocyte antigen). As a result, the members of some ethnic or racial groups may not have a safe and readily available CGT or RM product. This is why developers may consider partnering with stem cell registries, which strive to maintain a diverse donor base able to meet patient needs. These registries may also assist in determining donor eligibility criteria, collecting and transporting HSCs, and even selecting the most appropriate product for recipients.

## 6 Bringing cell and gene therapies and regenerative medicine products to Québec patients

**FIGURE 2:**
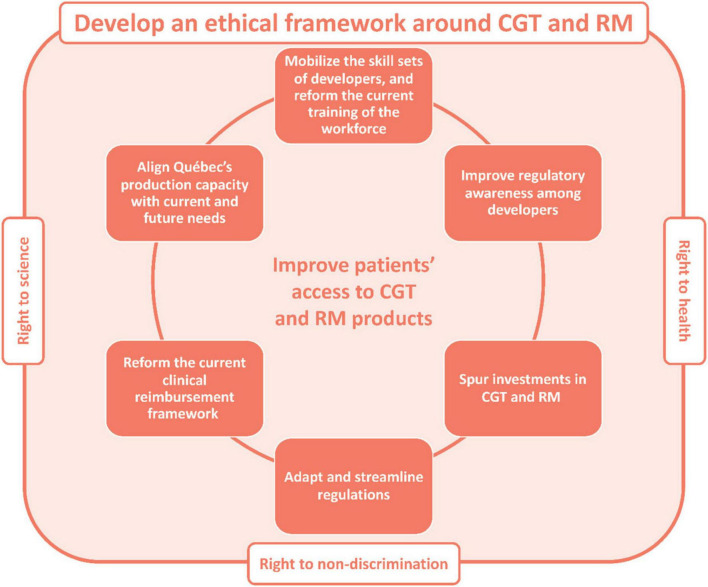
Proposed strategic orientations to improve patients’ access to CGT and RM products in Québec, Canada. CGT, cell and gene therapy; RM, regenerative medicine.

The solutions outlined above may be grouped under seven orientations which, together, provide guidance for bringing CGT and RM products to Québec patients (Figure 2). The first one is to develop an ethical framework around CGT and RM that will enable fair and timely access to these treatments for all. Second, governments could aim to spur private and public research investments in CGT and RM. Third, the skill sets of developers may be mobilized to foster the development and production of CGT and RM products, and the training of Québec’s workforce could be better aligned with industry and population needs to facilitate the industrialization of the sector, with the aim of reducing production costs and making these expensive technologies more accessible to patients. Fourth, Québec could lay out a plan to ensure that the province’s domestic production capacity is aligned with current and future needs in CGT and RM products, considering the rapidly evolving landscape of CGT and RM. Fifth, regulatory awareness may be improved among developers through outreach approaches (initiated by the regulator) and early consultations (initiated by developers). Sixth, the regulations governing the development of CGT and RM products could be streamlined and further adapted to the needs of these emerging products. Lastly, ongoing efforts to reform the current reimbursement framework may be continued, considering the specificities of Québec’s public, single-payer health care system.

To be sure, these high-level orientations should be implemented after seeking the input of patients. Although it fell out of the scope of our symposium, a patient partnership approach will be crucial to capturing the patient’s perspective on CGT and RM. Historically, the role of patients in health care improvement has been somewhat passive, e.g., as participants in clinical trials. However, patients can play a more active role if they are included as stakeholders, as shown by initiatives in Canada and elsewhere (40–43). Indeed, their experience can enrich the discussions held by public health institutions, pharmaceutical and biotechnology companies, and various government agencies, thereby improving the health care system and the health of the population. Hence, an important next step will be to integrate the patients’ perspective into the discussions, which should further help guide the development of policies to bring new CGT and RM products to patients.

## 7 Discussion

Together, these productive discussions pave the way to the implementation of new policies that will help promote the right to science, health, and non-discrimination in Québec. Although the scope of our symposium was limited to Québec, other jurisdictions may experience similar challenges, in which case many of the solutions discussed herein may be relevant elsewhere in Canada and worldwide. Conversely, Québec’s ecosystem may benefit from knowing more about the experience of other jurisdictions with similar health care systems, as in some European countries. Thus, we invite other jurisdictions to share their own challenges and opportunities in CGT and RM in the literature.
