# Correction: Harnessing the promises of cell therapy, gene therapy, and regenerative medicine in Québec, Canada

**DOI:** 10.3389/fmed.2025.1710876

**Published:** 2025-10-17

**Authors:** Jean-Sébastien Delisle, Diane Fournier, Kim Santerre, Samuel Rochette, Ma'n H. Zawati, Maude Dumont-Lagacé, Simon Turcotte, Jason Robert Guertin, Patrick Vermette, Jacques P. Tremblay, Maxime Parisotto, Christian Beauséjour, Jean-François Gélinas, Louisa Petropoulos, Natasha Kekre, Stéphanie Michaud, Mélanie Dieudé

**Affiliations:** ^1^Centre de recherche de l'Hôpital Maisonneuve-Rosemont, Montreal, QC, Canada; ^2^Institut d'hématologie, oncologie et thérapie cellulaire (iHOT), Hôpital Maisonneuve-Rosemont, Montreal, QC, Canada; ^3^Département de médicine, Faculté de médecine, Université de Montréal, Montreal, QC, Canada; ^4^Québec Cell, Tissue and Gene Therapy Network, Montreal, QC, Canada; ^5^Transfusion Medicine, Héma-Québec, Montreal, QC, Canada; ^6^Centre of Genomics and Policy, Department of Human Genetics, Faculty of Medicine and Health Sciences, McGill University, Montreal, QC, Canada; ^7^Borealis Medical Writing Inc., Montreal, QC, Canada; ^8^Cancer Axis, Centre de recherche du Centre hospitalier de l'Université de Montréal, Montreal, QC, Canada; ^9^Department of Hepatopancreatobiliary Surgery and Liver Transplantation Service, Centre hospitalier de l'Université de Montréal, Montreal, QC, Canada; ^10^Axe santé des populations et pratiques optimales en santé, Centre de recherche du Centre hospitalier universitaire de Québec, Université Laval, Québec City, QC, Canada; ^11^Département de médecine sociale et préventive, Faculté de médecine, Université Laval, Québec City, QC, Canada; ^12^Centre de recherche en organogénèse expérimentale de l'Université Laval/LOEX, Québec City, QC, Canada; ^13^Laboratoire de bio-ingénierie et de biophysique de l'Université de Sherbrooke, Department of Chemical and Biotechnological Engineering, Université de Sherbrooke, Sherbrooke, QC, Canada; ^14^Faculté de Médecine et des Sciences de la santé, Centre de Recherche du Centre Hospitalier Universitaire de Sherbrooke, Sherbrooke, QC, Canada; ^15^Centre de Recherche du CHU de Québec-Université Laval, Québec City, QC, Canada; ^16^Département de Médecine Moléculaire, Université Laval, Québec City, QC, Canada; ^17^adMare BioInnovations, Montreal, QC, Canada; ^18^Centre de Recherche du Centre hospitalier Universitaire Sainte-Justine, Montreal, QC, Canada; ^19^Département de Pharmacologie et Physiologie, Faculté de Médecine, Université de Montréal, Montreal, QC, Canada; ^20^Centre C3i Inc., Montreal, QC, Canada; ^21^Division of Hematology, Department of Medicine, The Ottawa Hospital, Ottawa, ON, Canada; ^22^BioCanRx, Ottawa, ON, Canada; ^23^Axe immunopathologie, Centre de recherche du Centre hospitalier de l'Université de Montréal, Montreal, QC, Canada; ^24^Département de microbiologie, Infectiologie et immunologie, Faculté de médecine, Université de Montréal, Montreal, QC, Canada; ^25^Héma-Québec, Medical Affairs and Innovation, Montreal, QC, Canada

**Keywords:** immune effector cell immunotherapy, research funding, economic evaluation, ethics, biological drug approval, cell and tissue manufacturing

Author Jean-François Gélinas was omitted as an author in the published article.

The authors apologize for this error and state that this does not change the scientific conclusions of the article in any way. All authors agreed to the correction.

The corrected author list appears below (there are no changes to the affiliation list):

Jean-Sébastien Delisle^1,2,3,4^^*^, Diane Fournier^5^^*^, Kim Santerre^4^, Samuel Rochette^5^, Ma'n H. Zawati^4.6^, Maude Dumont-Lagacé^5,7^, Simon Turcotte^4,8,9^, Jason Robert Guertin^4,10,11,12^, Patrick Vermette^4,13,14^, Jacques P. Tremblay^4,15,16^, Maxime Parisotto^17^, Christian Beauséjour^4,18,19^, Jean-François Gélinas^4,20^ Louisa Petropoulos^20^, Natasha Kekre^21^, Stéphanie Michaud^22^, Mélanie Dieudé^23,24,25^^*^.

In the published article, the author contribution and conflict of interest statements were missing the information of author Jean-François Gélinas, who was erroneously excluded from the author list.

A correction has been made to the section Author Contributions to add the contributions of Jean-François Gélinas. The added sentence appears below:

“J-FG: Data curation, Formal analysis, Writing – review and editing.”

A correction has also been made to the section Conflict of Interest to add information on author Jean-François Gélinas. This sentence previously stated :

“LP was employed by Centre C3i Inc.”

The corrected sentence appears below:

“J-FG is and LP was employed by Centre C3i Inc.”

The original article has been updated.

